# Antibody-coupled monolithic silica microtips for highthroughput molecular profiling of circulating exosomes

**DOI:** 10.1038/srep06232

**Published:** 2014-08-29

**Authors:** Koji Ueda, Nobuhisa Ishikawa, Ayako Tatsuguchi, Naomi Saichi, Risa Fujii, Hidewaki Nakagawa

**Affiliations:** 1 Division of Biosciences, Functional Proteomics Center, Graduate School of Frontier Sciences, the University of Tokyo, Tokyo, Japan; 2 Laboratory for Genome Sequencing Analysis, Center for Integrated Medical Sciences, RIKEN, Tokyo, Japan; 3 Department of Molecular and Internal Medicine, Hiroshima University, Hiroshima, Japan

## Abstract

Exosome-mediated signal transportation plays a variety of critical roles in cancer progression and metastasis. From the aspect of cancer diagnosis, circulating exosomes are ideal resources of biomarkers because molecular features of tumor cells are transcribed on them. However, isolating pure exosomes from body fluids is time-consuming and still major challenge to be addressed for comprehensive profiling of exosomal proteins and miRNAs. Here we constructed anti-CD9 antibody-coupled highly porous monolithic silica microtips which allowed automated rapid and reproducible exosome extraction from multiple clinical samples. We applied these tips to explore lung cancer biomarker proteins on exosomes by analyzing 46 serum samples. The mass spectrometric quantification of 1,369 exosomal proteins identified CD91 as a lung adenocarcinoma specific antigen on exosomes, which was further validated with CD9-CD91 exosome sandwich ELISA measuring 212 samples. Our simple device can promote not only biomarker discovery studies but also wide range of omics researches about exosomes.

Lung cancer is the leading cause of cancer-related mortality worldwide, accounting for 1,475,117 deaths in 2011 (Global Health Observatory Data Repository, World Health Organization). The high mortality is mainly attributable to a late-stage diagnosis and the lack of effective treatments. Indeed, by means of current cancer screening tests, only 30% of patients are diagnosed at an early disease stage and present surgically resectable tumors^1^. Therefore development of novel biomarkers and establishment of blood-based early detection system for lung cancer is crucial in order to improve clinical outcome and overall survival rate.

Recently biological significance and clinical utility of exosomes have been extensively discussed. Particularly contribution of tumor-derived exosomes to the formation of metastatic microenvironments is one of the most fundamental functions of them, which would provide a better understanding for cancer metastasis and even new therapeutic strategies to prevent metastasis^2^,^3^,^4^. Exosome-mediated delivery of therapeutic RNAs has been already in a pioneering stage for cancer treatment^5^,^6^. In the field of cancer diagnosis, exosomes are also fascinating targets for biomarker discovery due to their molecular characteristics^7^,^8^,^9^. In principle, a set of molecules expressed in original solid tumor cells would be detectable as exosomal components in blood circulation. Despite the theoretical feasibility of exosomal biomarkers, difficulties in exosome isolation from biological fluids have significantly hindered effective discovery of biomarker candidates. In fact, although ultracentrifugation-based methods are the most common strategies to isolate exosomes from serum samples^10^, the reproducibility, processing time, and purity are not appropriate for biomarker screening studies dealing with a lot of clinical samples quantitatively^11^.

In the present study, we established an antibody-assisted exosome purification tips by immobilizing anti-CD9 antibody to Mass Spectrometric Immunoassay (MSIA) monolith pipette tips. This multi-channeled platform effectively streamlined proteome-wide mass spectrometric profiling of serum exosomes and allowed accurate statistical identification of lung cancer-specific exosomal proteins. We further constructed exosome sandwich ELISA assays for large-scaled replication study to validate screening reliability for an identified exosome surface antigen CD91.

## Results

### Isolation of serum exosomes by anti-CD9-MSIA tips

**Figure 1:**
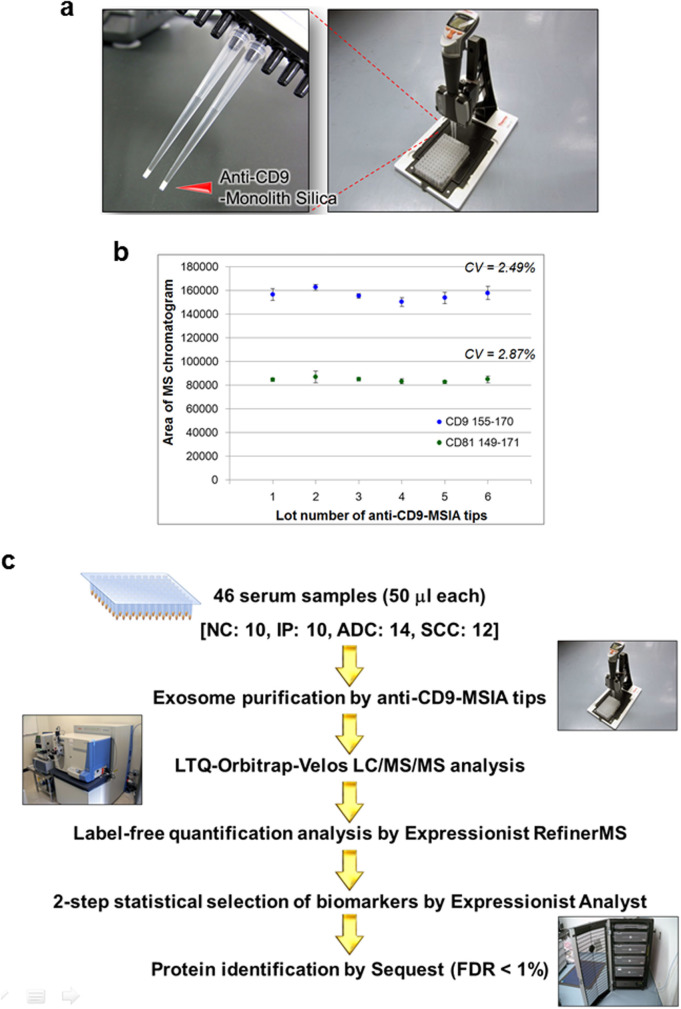
Schematic view of exosomal biomarker discovery workflow. (a) Magnified picture of anti-CD9 MSIA tips (left) and a dedicated holding fixture (right). Pictures were taken by authors. (b) Exosome fractions were purified from a pooled serum sample using 6 independent anti-CD9-MSIA tips and analyzed by LC/MS/MS in triplicated measurements. The coefficient of variation (CV) of peak intensities corresponding to CD9 155-170 peptide (GLAGGVEQFISDICPK, m/z = 845.9266) or CD81 149-171 peptide (TFHETLDccGSSTLTALTTSVLK, m/z = 848.0733) was shown. (c) Exosomes were isolated from 46 serum samples by anti-CD9 antibody-coupled monolith tips (anti-CD9-MSIA tips) on 12-channel automatic pipetting platform. The enriched exosome fractions were analyzed by LC/MS/MS and subjected to label-free quantification analysis by RefinerMS software on the Expressionist proteome server system. The quantified peptides underwent 2-step statistical analysis, composed of ANOVA and feature elimination method, and finally extracted biomarker candidate peptides were identified with Sequest database search. The identification threshold was set at false discovery rate (FDR) < 1%.

To perform reproducible and high-purity separation of exosomes from serum, we employed the antibody-immobilized low back pressure monolithic tips on automated 12-channel pipette system (Figure 1a), which allowed 30 minutes isolation of exosomes from 12 serum samples simultaneously. Here we selected a tetraspanin molecule CD9 as a target of exosome-capturing antibody due to its strong expression on the surface of exosomes secreted from diverse cell types^12^. In order to evaluate the reproducibility of anti-CD9-MSIA tips, exosomes were purified from a pooled serum sample using 6 independent tips and analyzed by LC/MS/MS in triplicated measurements (Figure 1b). The coefficient of variation (CV) of peak area corresponding to CD9 155-170 peptide (GLAGGVEQFISDICPK, m/z = 845.9266) or CD81 149-171 peptide (TFHETLDCCGSSTLTALTTSVLK, m/z = 848.0733), which was also known as a typical exosome marker molecule, was 2.49% or 2.87%, respectively, indicating that the error level in relative quantification analysis was small enough for reliable biomarker identification. Then we next isolated serum exosomes from 10 normal controls (NC), 10 interstitial pneumonia patients (IP), 14 lung adenocarcinoma patients (ADC), and 12 lung squamous cell carcinoma patients (SCC) using anti-CD9-MSIA tips. Purified exosomes were individually analyzed by LC/MS/MS system and subjected to statistical analysis as shown in Figure 1c.

### Proteome-wide overview of human serum exosomes

**Figure 2:**
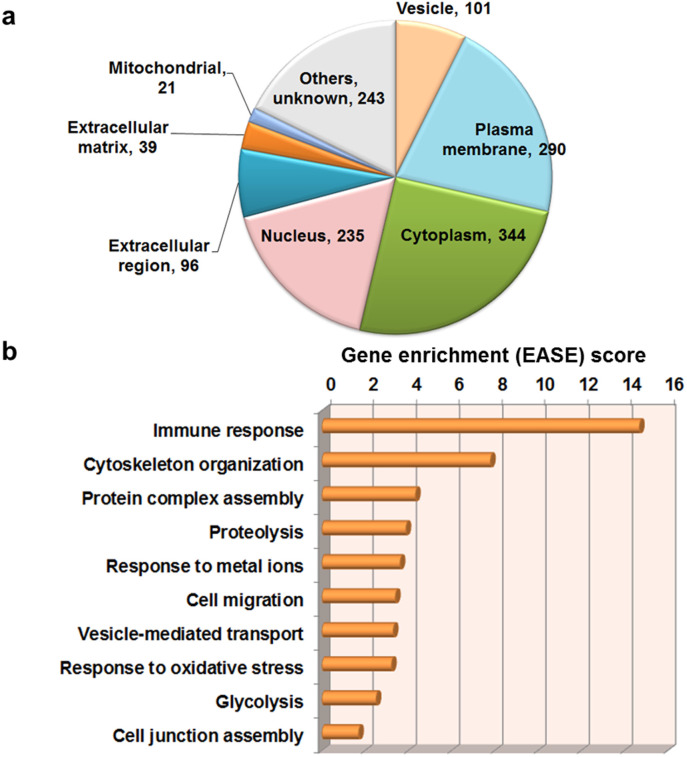
Proteome-wide overview of 1,369 identified exosomal proteins. (a) Distribution of protein subcellular localization was shown in a pie chart. (b) The Fisher Exact Statistics in DAVID system was used for functional annotation clustering analysis. The 10 enriched functions detected in 1,369 exosomal proteins were shown with Expression Analysis Systematic Explorer (EASE) scores.

The LC/MS/MS analysis of 46 serum samples (Table 1) and subsequent Sequest database search identified 1,369 non-redundant proteins (FDR < 1%, Supplementary Table 1). To assess the purity of anti-CD9-MSIA tip eluates, identified proteins were classified according to subcellular localizations (Figure 2a). The Cellular Component distribution by DAVID GO analysis illustrated highly-enriched 701 intracellular proteins (51.2%) and 290 plasma membrane proteins (21.2%), whereas only 135 extracellular (secreted) proteins (9.8%) were identified. These values clearly represented efficient enrichment of exosomes bearing original cell-derived cellular components. Importantly, most of serum abundant proteins such as albumin and IgG were effectively washed out during MSIA purification steps, which often hindered the sensitive detection of minor exosomal proteins. Moreover to elucidate physiological functions of serum exosomes, Expression Analysis Systematic Explorer (EASE) scores were calculated^13^,^14^ (Figure 2b). This functional estimation suggested the possible association of serum exosomes with immune regulations, cell-to-cell interactions, and stimulation responses, in addition to vesicle transport. These data would contribute to new revelations about the biological functions of not only tumor-derived exosomes but also normal exosomes.

### Statistical identification of exosomal biomarkers for lung cancer

**Figure 3:**
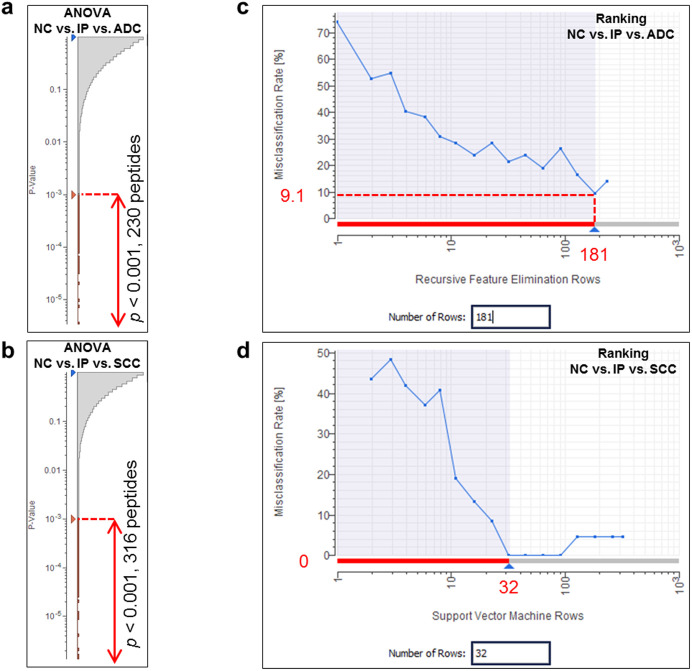
Two-step statistical selection of biomarker candidates. For the first stage, 3-group ANOVA was performed to compare NC, IP, and ADC groups (a) or NC, IP, and SCC groups (b). Peptides satisfying the criterion *p* < 0.001 were used for the second Ranking selection. To calculate the minimum set of biomarkers which could provide the minimum misclassification rate, cross validation-based support vector machine-recursive feature elimination (SVM-RFE) method was used for comparison of NC, IP, and ADC groups (c). Similarly, SVM-SVM method was employed for comparison of NC, IP, and SCC groups (d). The number of selected biomarker candidates and the misclassification rate were shown. NC, normal control; IP, interstitial pneumonia; ADC, adenocarcinoma; SCC, squamous cell carcinoma.

**Figure 4:**
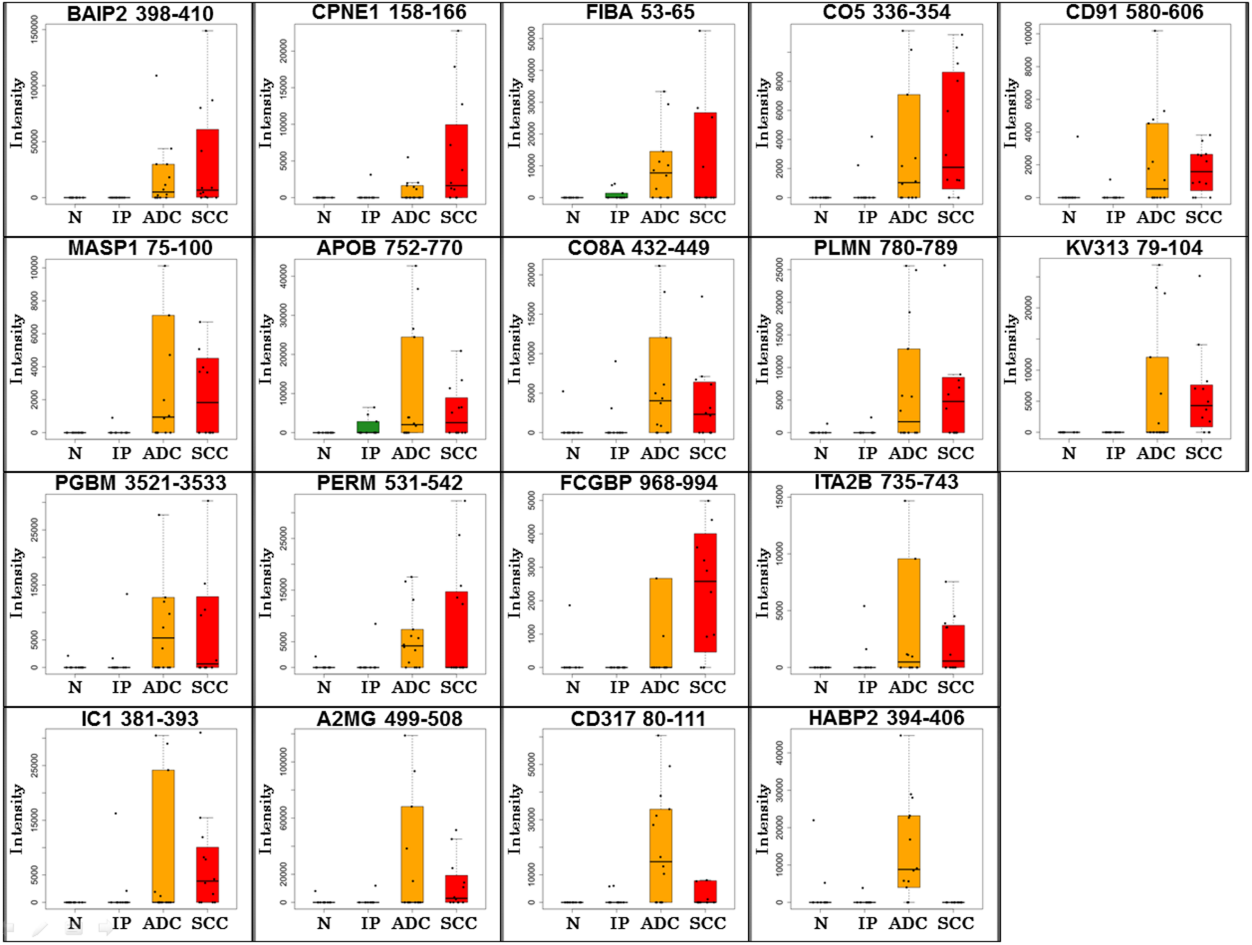
Identified 18 exosomal biomarker candidates. The LC/MS/MS signal intensities for 18 biomarker candidates acquired from 46 cases were displayed in box plots. The UniProtKB entry protein names accompanied with amino acid numbers were shown over the box plots. N, normal control; IP, interstitial pneumonia; ADC, adenocarcinoma; SCC, squamous cell carcinoma.

**Figure 5:**
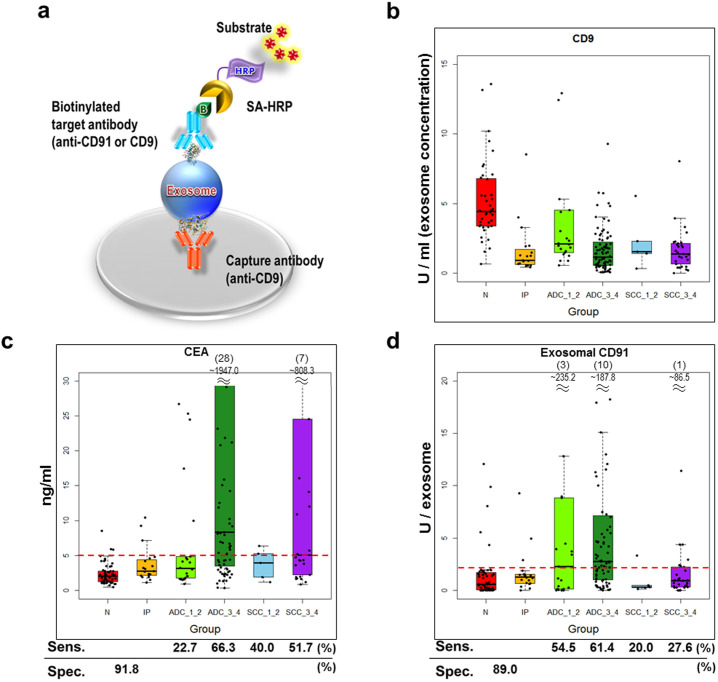
Exosome sandwich ELISA-based validation experiment for CD91. (a) Principle of exosome sandwich ELISA. SA-HRP, streptavidin-horseradish peroxidase. Using 212 independent serum samples, exosomal CD91 and CEA concentrations were measured. (b) Serum exosome concentrations were determined by CD9-CD9 sandwich ELISA. (c) CEA concentrations were measured by commercial ELISA kits. (d) Exosomal CD91 concentrations were determined by CD9-CD91 sandwich ELISA. The values were normalized with exosome concentrations calculated in (b). Red lines indicate the cut-off values for CEA at 5.0 ng/ml (c) or exosomal CD91 at 2.04 U/exosome (d). The sensitivity (Sens.) for each lung cancer sub-group and specificity (Spec.) were shown below the box plots. N, normal control; IP, interstitial pneumonia; ADC_1_2, stage-I, II adenocarcinoma; ADC_3_4, stage-III, IV adenocarcinoma; SCC_1_2, stage-I, II squamous cell carcinoma; SCC_3_4, stage-III, IV squamous cell carcinoma.

The label-free quantification analysis on the Expressionist RefinerMS module (Figure 1c and Supplementary Figure 1) quantified 113,582 non-redundant peptides from 46 serum samples. In the first statistical selection, 3-group ANOVA was used to roughly extract signature peptides specific to ADC patients (230 peptides, *p* < 0.001) or SCC patients (316 peptides, *p* < 0.001) as shown in Figure 3a or 3b, respectively. Here, to identify lung cancer specific exosomal biomarkers which don't react with inflammatory lung diseases such as interstitial pneumonia, IP patients were also considered as a non-cancer control group. For the second stage, cross validation-based feature elimination method was employed to compute the minimum biomarker sets which provided the least misclassification rates. Here support vector machine recursive feature elimination (SVM-RFE) method or SVM-SVM method defined 181 or 32 peptides as final candidate biomarker sets demonstrating 90.9% or 100% true prediction rate for ADC or SCC patients group (Figure 3c or 3d, respectively). By referring to protein identification data, 20 peptides derived from 18 proteins were identified (Figure 4 and Table 2). Among them, CD91, Integrin alpha-IIb, and CD317 were selected as favorable exosomal biomarker candidates because their expected localization was on the surface of exosomes and could be measured by exosome sandwich ELISA system (Figure 5a).

### Validation experiment for CD91 by exosome sandwich ELISA

**Figure 6:**
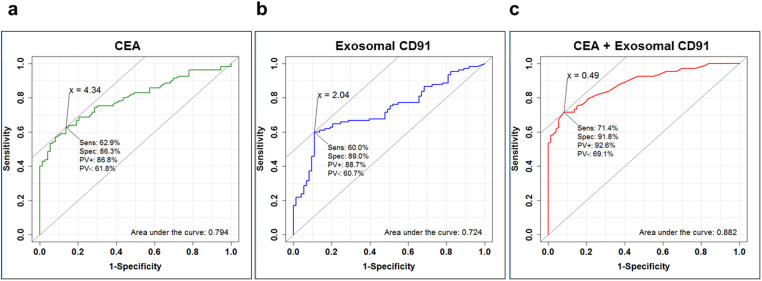
ROC curve analysis for exosomal CD91 and CEA. ROC curves for CEA (a), exosomal CD91 (b), and logistic regression-based combination marker CEA + exosomal CD91 (c) were depicted by R. The diagnostic efficiencies between NC + IP (n = 73) and lung ADC patients (n = 105) were evaluated. The cut-off value was set at the point whose distance from the (sensitivity, specificity) = (1, 1) reached the minimum. The sensitivity (Sens), specificity (Spec), positive predictive value (PV+), negative predictive value (PV-), and area under the curve (AUC) were shown on each graph.

To assess the quantitative reproducibility of the label-free quantification results in our single-run screening analysis, as well as the clinical usefulness of a candidate biomarkers, we conducted further validation study by exosome sandwich ELISA using 212 independent serum samples (Table 1). Among three candidate biomarker proteins, we eventually succeeded to obtain good antibody and construct ELISA assay only for CD91. In this assay, we utilized anti-CD9 antibody as an exosome-capture antibody and biotinylated anti-CD9 or biotinylated anti-CD91 antibody as a detection antibody (Figure 5a). Since serum exosome concentrations, measured by CD9-CD9 sandwich ELISA, had drastic individual variability (Figure 5b), the measurements of CD9-CD91 sandwich ELISA were normalized by exosome concentrations (denoted by U/exosome in Figure 5c). We also tested a classical clinical biomarker CEA in the same sample set (Figure 5d) and compared the diagnostic efficacy to that of exosomal CD91. When we set the cut-off value at 2.04 U/exosome for exosomal CD91 and 5.0 ng/ml for CEA, exosomal CD91 showed significantly higher sensitivity for detecting stage-I, II ADC patients (54.5%) compared with CEA (22.7%), while the detection power of exosomal CD91 in stage-III, IV ADC patients (61.4%) was similar with that or CEA (66.3%). The false positive rate of exosomal CD91 in the control group (NC and IP, n = 73) were 11.0%, while that of CEA was 8.2%. These results indicated that exosomal CD91 possessed better potential for the early detection of lung ADC compared with CEA. On the other hand, exosomal CD91 might not be useful in the detection of SCC patients. In addition, we constructed a logistic regression model to evaluate the advantage of a combination of exosomal CD91 and CEA. The ROC curve analysis using control samples (NC and IP, n = 73) and all ADC samples (n = 105) in Figure 6a–c revealed that the combination biomarker effectively improved all of sensitivity (71.4%), specificity (91.8%), and area under the curve (0.882), compared with each single biomarker. Additionally, effects of age or gender on exosomal CD91 concentrations were assessed. The results indicated that exosomal CD91 was affected by neither age (R^2^ = 0.0536, Supplementary Figure 2a) nor gender (*p* = 0.299, Supplementary Figure 2b). These results suggested that exosomal CD91 was an independent predictor of lung ADC which could complement diagnostic power of CEA.

## Discussion

In recent years, physiological roles of secreted microvesicles, including exosomes, were actively studied mainly using cell culture supernatants (CCS). However studies on body fluid exosomes have been still at the stage of seeking appropriate technologies which allow high-purity isolation of exosomes. In fact, ultracentrifugal sedimentation methods, the most common techniques to purify exosomes^15^, can not remove large size proteins (e.g. α2-macroglobulin, IgM, complement factors, and so on), aggregated proteins, circulating proteasome^16^,^17^, and vault ribonucleoprotein particles^18^ in serum because they have similar diameters (40 ~ 100 nm) and sedimentation coefficient to exosomes. Owing to these major contaminants, serum-derived proteins often occupy a great part of identified proteins by LC/MS/MS analysis of ultracentrifugal sedimentation samples (data not shown). In addition to the purity issue, throughput and reproducibility are also critical factors for the development of exosome isolation methods because biomarker screening studies or therapeutic target discovery studies usually deal with lots of clinical specimens. To satisfy these technological requirements, we established a rapid, reproducible, and high-quality isolation device by integrating exosome capture antibody, low pressure monolith tips, and 12-well automatic pipet. Since the MSIA tips are also compatible with commercial 96-well automatic pipetting workstations, our method is applicable for larger (> 100 cases) sample set studies. In the present study, we used anti-CD9 antibody to capture exosomes in both MSIA tip devices and exosome sandwich ELISA system. Although CD9 is one of the most well-known exosome markers, the expression level of CD9 might vary by tissues^19^ or disease state^20^,^21^. These facts indicate that a single use of anti-CD9 antibody could provide proteome profiles for only a limited proportion of serum exosomes. Therefore effective combination of antibodies specific to various exosome markers (e.g. CD63 or CD81) would improve the comprehensiveness of exosome profiling studies.

From the proteome-wide biomarker screening of 46 serum samples, we eventually identified 18 biomarker candidate proteins in Figure 4 and Table 2. Concerning subcellular localizations of them, 6 proteins express in intracellular regions (BAIP2 in peripheral membrane area; copine-1 on vesicle membrane; myeloperoxidase in lysosome; CD91, integrin alpha-IIb, and CD317 on plasma membrane), however, other 13 proteins were known as major serum proteins. Although the number of identified extracellular proteins was small as shown in Figure 2a, the purification efficiency of anti-CD9-MSIA tip was still considered incomplete. In order for further improvement of this device, we're now optimizing the coating agents for monolith polymer in the tips to minimize non-specific binding of serum proteins. Because the amount of exosome-derived proteins is indeed ultratrace level compared to serum major proteins, the improvement of exosome purification efficiency could significantly increase the chances of detecting minor exosomal antigens associated with cancer development or progression. More effective and inexpensive exosome isolation method could also promote development of mass spectrometric multiplexed biomarker diagnosis measuring two or more exosomal biomarkers simultaneously by MRM/SRM assay.

In this report, we showed that CD91 expression was significantly elevated on exosomes in especially lung ADC patients' sera. The detection power for limited number of early stage patients (n = 22) was also higher than existing biomarker CEA (Figure 5c and 5d), but so far we can conclude that the exosomal CD91 assay could detect at least a large cancer burden. For functional consideration of CD91, this molecule is a type-1 transmembrane receptor which mediates ligand endocytosis in clathrin-coated pits and cargo trafficking to lysosomes^22^,^23^. Another fundamental role of CD91 is reported as a signaling receptor regulating cytokine secretion, phagocytosis and migration of cells in the immune system^24^,^25^. Notably, with regard to cancer, no or very low expression of CD91 in lung cancer cells was observed in tissues from ADC patients with poor clinical outcome, while strong staining patterns of CD91 were observed in stromal cells surrounding cancer cells from 94/111 ADC patients^26^. Combined with our results, high level of serum CD91-expressing exosomes would be originally secreted from stromal cells surrounding lung cancer cells. Since construction of tumor microenvironments is one of the most well-established functions of exosomes^3^, inhibiting production or function of CD91-positive exosomes might lead to suppression of lung cancer progression. Therefore inhibitors of CD91, which interfere ligand binding, such as the receptor-associated protein (RAP), suramin, α2-macroglobulin, and lactoferrin^27^ would be useful for investigating the association of exosomal CD91 and lung cancer. In contrast to CD91, another candidate of exosomal biomarker CD317 was already confirmed as a potential therapeutic target for lung cancer^28^. Due to its nature of lung cancer specific expression, chimeric or humanized antibody drugs were developed and tested in clinical trials^29^. This evidence indicated that the lung ADC cells directly release high level of CD317-positive exosomes in blood circulation.

Thus in addition to identification of biomarker candidates, our anti-CD9 MSIA tips can provide diverse novel knowledge about human exosomes. More comprehensive information will be also obtained by replacing the epitope of immobilized antibody with other exosomal surface antigens such as CD63, CD81, tetraspanin-9, or tetraspanin-14.

## Methods

### Serum samples

Serum samples from lung cancer patients (n = 165), interstitial pneumonia patients (n = 29), and normal controls (n = 64) were collected in Hiroshima University Hospital within the same period. All samples were collected from untreated patients at the initial visit to hospital. The nonanticoagulated blood samples were collected and allowed to clot at room temperature for 1–2 hours. Sera were then separated by centrifugation at 1500 rpm for 15 min and stored frozen at −80°C. Written informed consents were obtained from all participants. This study was approved by The Ethical Committee of RIKEN (Approval code: Yokohama H20-12) and The Ethical Committee of Hiroshima University Hospital. All experiments were performed in accordance with relevant guidelines and regulations.

### Exosome purification by anti-CD9 MSIA tips

All procedures were performed on Novus-i 12-channel electronic pipettes and adjustable pipette stand (Thermo Fisher Scientific, Waltham, Massachusetts, USA). The MSIA D.A.R.T.'s, Protein G tips (Thermo Fisher Scientific) were equilibrated with 300 µl PBS × 10 cycles prior to 25 µl × 100 cycles of immobilization of 1 µg anti-CD9 antibody (provided by Shionogi & Co., Ltd., Osaka, Japan) in 50 µl PBS. After crosslinking with 0.25 mM BS3 (Thermo Fisher Scientific) in PBS (100 µl × 100 cycles), reaction was quenched with 50 mM ethanolamine-HCl (pH 8.0) (100 µl × 100 cycles). Following 300 µl × 10 cycles of equilibration in PBS, the anti-CD9-MSIA tips were incubated with 350 µl of seven-fold diluted serum samples by 300 µl × 100 cycles pipetting. Tips were washed three times by 300 µl × 25 cycles pipetting in PBS. Finally captured exosomes were eluted by 20 µl × 100 cycles pipetting in 30 µl of [8M Urea, 50 mM ammonium bicarbonate] solution.

After reduction with 5 mM TCEP at 37°C for 30 minutes and alkylation with 25 mM iodoacetamide at room temperature for 45 minutes, samples were diluted 7 times with 50 mM ammonium bicarbonate. Proteins were digested by Immobilized Trypsin beads (Thermo Fisher Scientific) in a 96-well filter plate with continuous shaking at 37°C for 6 hours. Tryptic digests were desalted by Oasis HLB 96-well µElution Plate (Waters Corporation, Milford, Massachusetts, USA) and subjected to LC/MS/MS analysis.

### LC/MS/MS analysis

The dried peptide samples were resuspended in 2% acetonitrile with 0.1% trifluoroacetic acid and analyzed by LTQ-Orbitrap-Velos mass spectrometer (Thermo Fisher Scientific) combined with UltiMate 3000 RSLC nano-flow HPLC system (DIONEX Corporation, Sunnyvale, California, USA). Samples were separated on 75 μm × 150 mm C_18_ tip-column (Nikkyo Technos, Tokyo, Japan) using solvent A [0.1% formic acid] and solvent B [0.1% formic acid in acetonitrile] with multistep linear gradient of solvent B 6.4 to 30% for 95 minutes and 30 to 95% for 10 minutes at a flow rate 250 nl/min. The eluted peptides were ionized with the spray voltage 2000 V and MS data was acquired in a data-dependent fragmentation method in which the survey scan was acquired between m/z 400 to 1600 at the resolution 60,000 with automatic gain control (AGC) target value of 1.0 × 106 ion counts. The top-20 intense precursor ions in each survey scan were subjected to low resolution MS/MS acquisitions using normal CID scan mode with AGC target value of 5,000 ion counts in the linear ion trap.

The protein identification analysis was performed by SEQUEST database search on Proteome Discoverer 1.3 software (Thermo Fischer Scientific). The MS/MS spectra were searched against human protein database SwissProt 2013_03 (20,255 sequences) using search parameters as follows: Enzyme Name = Semitrypsin, Precursor Mass Tolerance = 10 ppm, Fragment Mass Tolerance = 0.8 Da, Dynamic Modification = Oxidation (Met), and Static Modification = Carbamidomethyl (Cys). False discovery rate of 0.01 and Peptide Rank of 1 were set for peptide identification filters. The gene ontology analysis was performed on EASE (Expression Analysis Systematic Explorer, version 2.0) (http://www.geneontology.org/) to compute the overrepresented functional categories in “Cellular Component,” and “Molecular Function”.

### Label-free quantification analysis on Expressionist server

The LC/MS/MS data set was loaded to Expressionist RefinerMS module (Genedata AG, Basel, Switzerland) for data processing and label-free quantification analysis. The whole workflow of RefinerMS software was shown in Supplementary Figure S1. The Spectrum Grid was set at every 10 data points on 2D MS chromatogram planes (x = m/z and y = RT). The Structure Removal was sequentially performed with RT = 2 scans and m/z = 6 points in the first and second Chemical Noise Subtraction, respectively, followed by the third Subtraction using RT Window = 500 scans and Quantile = 90%. After forth Subtraction with RT = 2 scans, signals with intensity < 2,000 were clipped off by Intensity Thresholding. Finally further Structure Removals with RT = 2 scans and m/z = 5 points were run as the fifth and sixth Chemical Noise Subtractions, respectively.

Then Chromatogram Grid was set at every 10 scans on noise-subtracted data, followed by Chromatogram RT Alignment using parameters: m/z Window = 11 points, RT Window = 11 scans, Gap Penalty = 1, RT Search Interval = 2 minutes, and Alignment Scheme = pairwise alignment based tree. Next, the Summed Peak Detection Activity detected the peaks on a temporarily-averaged chromatogram with parameters as follows: Summation Window = 20 scans, Overlap = 10, Minimum Peak Size = 6 scans, Maximum Merge Distance = 1 data points, Gap/Peak Ratio = 5, Method = curvature-based peak detection, Peak Refinement Threshold = 5, and Consistency Filter Threshold = 1. Finally Summed Isotope Clustering Activity grouped isotopic peaks derived from single molecule into an isotope cluster. Here parameters were used as follows: Minimum Charge = 1, Maximum Charge = 6, Maximum Missing Peaks = 0, First Allowed Gap Position = 10, Ionization = protonation, RT Tolerance = 0.1 minute, m/z Tolerance = 0.01 Da, and Minimum Cluster Size Ratio = 0.5.

### Statistical analysis on Expressionist Analyst

Two-step statistical selection was employed for the effective identification of biomarker set. In the first stage, 3-group ANOVA was performed to roughly extract the candidates showing significantly distinct expression level among three clinical groups (*p* < 0.001). Next the minimum combination of biomarkers which provided the best classification rate was estimated by the Support Vector Machine-Recursive Feature Elimination (SVM-RFE) Ranking method in the Expressionist Analyst module (Genedata AG). SVM-RFE is an iterative algorithm that works backward from an initial set of statistical features. As a classifier, SVM was used for inferring decision rules. RFE was used as a ranking method which iteratively dropped the 10% peptides with the lowest weights in each step. Finally a Line Plot visualizer was returned displaying the average misclassification rate. Each classifier was represented by a line and the optimal classifier and biomarker peptide set size for the chosen ranking method could be read off.

### Exosome sandwich ELISA assay

The 250 ng/well of anti-CD9 antibody was immobilized to Nunc MaxiSorp flat-bottom 96 well plate (Thermo Fischer Scientific). The blocking solution (150 µl/well of 5% BSA in PBS) was then added and incubated on the plate shaker at ambient temperature for 60 minutes. After 3 times wash with PBS, [5 µl serum + 95 µl PBS] or [30 µl serum + 70 µl PBS] was loaded to the upper 48 wells or the lower 48 wells, respectively. Following 5 hours incubation, plates were washed three times by PBS. The 100 µl/well of biotinylated anti-CD9 antibody (125 ng/ml) or biotinylated anti-CD91 antibody (500 ng/ml, Abcam, Cambridge, UK) in 1% BSA was loaded to the upper 48 wells or the lower 48 wells, respectively. After 60 minutes incubation, plates were washed three times with PBS and then covered with 100 µl/well of 1×HRP-Streptavidin (Abcam) in 1% BSA solution. After 30 minutes incubation, plates were washed three times with PBS and covered with 100 µl/well of TMB Ready Solution (Thermo Fischer Scientific). The reaction was stopped after 10 minutes incubation using 100 µl/well of 2N HCl. The OD at 450 nm was immediately measured. The concentration data from CD9-CD9 and CD9-CD91 ELISA assays were normalized with gradient curves (5, 10, 15, 20, and 25 µl) obtained from a common pleural effusion sample. Finally CD91 concentrations were normalized with exosome concentration calculated with CD9-CD9 ELISA above.

### Box plot analysis and ROC curve analysis

The intensities of mass spectrum peaks corresponding to candidate biomarker peptides were displayed by box plot using R algorithm. For each study the box represents the middle half of the distribution of the data points stretching from the 25^th^ percentile to the 75^th^ percentile. The line across the box represents the median. The lengths of the lines above and below the box are defined by the maximum and minimum data point values, respectively, that lie within 1.5 times the spread of the box. ROC curves were also depicted by R. The cut-off value was set at the point whose distance from the (sensitivity, specificity) = (1, 1) reached the minimum. The sensitivity (Sens), specificity (Spec), positive predictive value (PV+), negative predictive value (PV-), and are under the curve (AUC) were shown on each graph.

## Author Contributions

K.U. designed the study and developed the method. K.U., N.I., A.T., N.S. and R.F. performed experiments and analyzed data. K.U. and H.N. interpreted the results and wrote the manuscript.

## Supplementary Material

Supplementary Information Supplementary Figures 1, 2

Supplementary Information Supplementary Table 1

## Figures and Tables

**Table 1:** Clinical Information of serum samples

	Screening set (n = 46)				Validation set (n = 212)			
	NC	IP	ADC	SCC	NC	IP	ADC	SCC
No. of samples	10	10	14	12	54	19	105	34
Age (average)	44.7	71.6	61.5	65.3	40.2	65.7	64.4	68.8
Gender (female/male)	4/6	3/7	6/8	2/10	29/25	5/14	35/70	7/27
TNM stage								
I			0	0			12	5
II			0	0			10	0
III			4	5			27	18
IV			10	7			56	11

NOTE: NC, normal control; IP, interstitial pneumonia; ADC, adenocarcinoma; SCC, squamous cell carcinoma.

**Table 2:** 18 exosomal lung cancer biomarker candidates

m/z	z	Protein Descriptions	UniProtKB entry	Protein Accessions	Sequence	XCorr
713.401	2	Brain-specific angiogenesis inhibitor 1-associated protein 2	BAIP2	Q9UQB8	EGDLITLLVPEAR	3.22
579.285	2	Copine-1	CPNE1	Q99829	SDPFLEFFR	2.67
507.583	3	Fibrinogen alpha chain	FIBA	P02671	GLIDEVNQDFTNR	4.54
1047.638	2	Complement C5	CO5	P01031	LNLVATPLFLKPGIPYPIK	3.79
1011.136	3	Prolow-density lipoprotein receptor-related protein 1	CD91/LRP1	Q07954	DGIHNVEGVAVDWMGDNLYWTDDGPK	4.57
953.123	3	Mannan-binding lectin serine protease 1	MASP1	P48740	ETTDTEQTPGQEVVLSPGSFMSITFR	5.28
1027.069	2	Apolipoprotein B-100	APOB	P04114	ILGEELGFASLHDLQLLGK	5.58
1100.068	2	Complement component C8 alpha chain	CO8A	P07357	YNPVVIDFEMQPIHEVLR	5.22
619.324	2	Plasminogen	PLMN	P00747	FVTWIEGVMR	3.31
481.584	3	Basement membrane-specific heparan sulfate proteoglycan core protein	PGBM	P98160	IAHVELADAGQYR	3.52
670.872	2	Myeloperoxidase	PERM	P05164	IGLDLPALNMQR	3.30
1441.657	2	IgGFc-binding protein	FCGBP	Q9Y6R7	VPSSYAEALCGLCGNFNGDPADDLALR	4.49
512.332	2	Integrin alpha-IIb	ITA2B	P08514	IVLLDVPVR	3.21
1020.122	3	Ig kappa chain V-III region HIC	KV313	P18136	LEPEDFAVYYCQQYGSSPWTFGQGTK	4.62
742.352	2	Plasma protease C1 inhibitor	IC1	P05155	VTTSQDMLSIMEK	2.65
542.811	2	Alpha-2-macroglobulin	A2MG	P01023	GHFSISIPVK	3.09
1141.520	3	Bone marrow stromal antigen 2	CD317/BST2	Q10589	GFQDVEAQAATCNHTVMALMASLDAEKAQGQK	3.11
727.377	2	Hyaluronan-binding protein 2	HABP2	Q14520	LKPVDGHcALESK	2.95

NOTE: m/z, mass/charge; z, charge; XCorr, cross-correlation score from Sequest database search.

## References

[b1] Lehtio J. & De Petris L. Lung cancer proteomics, clinical and technological considerations. J Proteomics 73, 1851–1863 (2010). 20685322 10.1016/j.jprot.2010.05.015

[b2] Luga V. *et al.* Exosomes mediate stromal mobilization of autocrine Wnt-PCP signaling in breast cancer cell migration. Cell 151, 1542–1556 (2012). 23260141 10.1016/j.cell.2012.11.024

[b3] Peinado H. *et al.* Melanoma exosomes educate bone marrow progenitor cells toward a pro-metastatic phenotype through MET. Nature medicine 18, 883–891 (2012). 10.1038/nm.2753 PMC3645291 22635005

[b4] Kosaka N. *et al.* Neutral sphingomyelinase 2 (nSMase2)-dependent exosomal transfer of angiogenic microRNAs regulate cancer cell metastasis. J Biol Chem 288, 10849–10859 (2013). 23439645 10.1074/jbc.M112.446831 PMC3624465

[b5] Grapp M. *et al.* Choroid plexus transcytosis and exosome shuttling deliver folate into brain parenchyma. Nature communications 4, 2123 (2013). 10.1038/ncomms3123 23828504

[b6] El-Andaloussi S. *et al.* Exosome-mediated delivery of siRNA in vitro and in vivo. Nat Protoc 7, 2112–2126 (2012). 23154783 10.1038/nprot.2012.131

[b7] Sandoval M. *et al.* The glycolytic enzyme aldolase C is up-regulated in rat forebrain microsomes and in the cerebrospinal fluid after repetitive fluoxetine treatment. Brain research 1520, 1–14 (2013). 23688545 10.1016/j.brainres.2013.04.049

[b8] Tanaka Y. *et al.* Clinical impact of serum exosomal microRNA-21 as a clinical biomarker in human esophageal squamous cell carcinoma. Cancer 119, 1159–1167 (2013). 23224754 10.1002/cncr.27895

[b9] Khan S. *et al.* Plasma-derived exosomal survivin, a plausible biomarker for early detection of prostate cancer. PLoS One 7, e46737 (2012). 23091600 10.1371/journal.pone.0046737 PMC3473028

[b10] Lasser C. Identification and analysis of circulating exosomal microRNA in human body fluids. Methods in molecular biology 1024, 109–128 (2013). 23719946 10.1007/978-1-62703-453-1_9

[b11] Witwer K. W. *et al.* Standardization of sample collection, isolation and analysis methods in extracellular vesicle research. Journal of extracellular vesicles 2, 20360 (2013). 10.3402/jev.v2i0.20360 PMC3760646 24009894

[b12] Keller S., Sanderson M. P., Stoeck A. & Altevogt P. Exosomes: from biogenesis and secretion to biological function. Immunology letters 107, 102–108 (2006). 17067686 10.1016/j.imlet.2006.09.005

[b13] Ford G., Xu Z., Gates A., Jiang J. & Ford B. D. Expression Analysis Systematic Explorer (EASE) analysis reveals differential gene expression in permanent and transient focal stroke rat models. Brain research 1071, 226–236 (2006). 16409990 10.1016/j.brainres.2005.11.090

[b14] Tan P. K. *et al.* Evaluation of gene expression measurements from commercial microarray platforms. Nucleic acids research 31, 5676–5684 (2003). 14500831 10.1093/nar/gkg763 PMC206463

[b15] Taylor D. D., Zacharias W. & Gercel-Taylor C. Exosome isolation for proteomic analyses and RNA profiling. Methods in molecular biology 728, 235–246 (2011). 21468952 10.1007/978-1-61779-068-3_15

[b16] Sixt S. U. & Dahlmann B. Extracellular, circulating proteasomes and ubiquitin - incidence and relevance. Biochimica et biophysica acta 1782, 817–823 (2008). 18602990 10.1016/j.bbadis.2008.06.005

[b17] Egerer K. *et al.* Circulating proteasomes are markers of cell damage and immunologic activity in autoimmune diseases. The Journal of rheumatology 29, 2045–2052 (2002). 12375310

[b18] Kedersha N. L., Heuser J. E., Chugani D. C. & Rome L. H. Vaults. III. Vault ribonucleoprotein particles open into flower-like structures with octagonal symmetry. The Journal of cell biology 112, 225–235 (1991). 1988458 10.1083/jcb.112.2.225 PMC2288824

[b19] Huang C. *et al.* MRP-1/CD9 and KAI1/CD82 expression in normal and various cancer tissues. Int J Oncol 11, 1045–1051 (1997). 21528303 10.3892/ijo.11.5.1045

[b20] Miyake M. *et al.* Motility related protein 1 (MRP-1/CD9) expression: inverse correlation with metastases in breast cancer. Cancer Res 55, 4127–4131 (1995). 7664290

[b21] Sauer G. *et al.* Progression of cervical carcinomas is associated with down-regulation of CD9 but strong local re-expression at sites of transendothelial invasion. Clin Cancer Res 9, 6426–6431 (2003). 14695144

[b22] Strickland D. K., Gonias S. L. & Argraves W. S. Diverse roles for the LDL receptor family. Trends in endocrinology and metabolism: TEM 13, 66–74 (2002). 11854021 10.1016/s1043-2760(01)00526-4

[b23] Weaver A. M., McCabe M., Kim I., Allietta M. M. & Gonias S. L. Epidermal growth factor and platelet-derived growth factor-BB induce a stable increase in the activity of low density lipoprotein receptor-related protein in vascular smooth muscle cells by altering receptor distribution and recycling. J Biol Chem 271, 24894–24900 (1996). 8798766 10.1074/jbc.271.40.24894

[b24] Yoon C. *et al.* Low-density Lipoprotein Receptor-related Protein 1 (LRP1)-dependent Cell Signaling Promotes Axonal Regeneration. J Biol Chem 288, 26557–26568 (2013). 23867460 10.1074/jbc.M113.478552 PMC3772203

[b25] Stiles T. L. *et al.* LDL receptor-related protein-1 is a sialic-acid-independent receptor for myelin-associated glycoprotein that functions in neurite outgrowth inhibition by MAG and CNS myelin. Journal of cell science 126, 209–220 (2013). 23132925 10.1242/jcs.113191 PMC3603516

[b26] Meng H. *et al.* Stromal LRP1 in lung adenocarcinoma predicts clinical outcome. Clin Cancer Res 17, 2426–2433 (2011). 21325077 10.1158/1078-0432.CCR-10-2385 PMC3079007

[b27] Vassiliou G., Benoist F., Lau P., Kavaslar G. N. & McPherson R. The low density lipoprotein receptor-related protein contributes to selective uptake of high density lipoprotein cholesteryl esters by SW872 liposarcoma cells and primary human adipocytes. J Biol Chem 276, 48823–48830 (2001). 11604390 10.1074/jbc.M103954200

[b28] Wang W. *et al.* HM1.24 (CD317) is a novel target against lung cancer for immunotherapy using anti-HM1.24 antibody. Cancer immunology, immunotherapy : CII 58, 967–976 (2009). 10.1007/s00262-008-0612-4 PMC11030068 18979097

[b29] Wang W. *et al.* Chimeric and humanized anti-HM1.24 antibodies mediate antibody-dependent cellular cytotoxicity against lung cancer cells. Lung cancer 63, 23–31 (2009). 18524412 10.1016/j.lungcan.2008.04.009

