# Supplementary material for: Antibody-coupled monolithic silica microtips for highthroughput molecular profiling of circulating exosomes
Source: Sci Rep. 2014 Aug 29;4:6232. doi: 10.1038/srep06232 (PMC4148700; doi:10.1038/srep06232)
Supplement: Supplementary Information — Supplementary Figures 1, 2 [file srep06232-s1.doc]

**Antibody-coupled monolithic silica microtips for highthroughput molecular profiling of circulating exosomes**

Koji Ueda, Nobuhisa Ishikawa, Ayako Tatsuguchi, Naomi Saichi, Risa Fujii, and Hidewaki Nakagawa

**Supplementary Figure 1**

Processing workflow of Expressionist proteomics quantification system

**Supplementary Figure 2**

Effect of age or gender on exosomal CD91 concentrations

**Supplementary Figure 1**


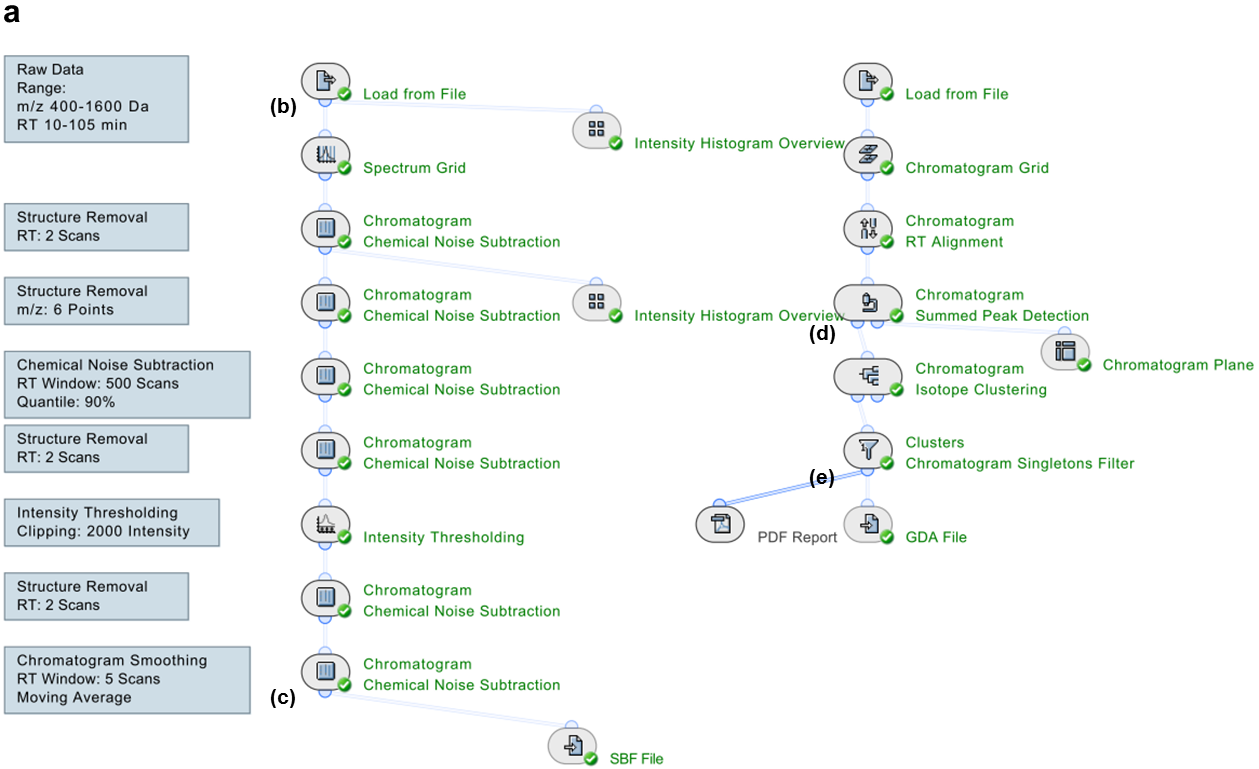


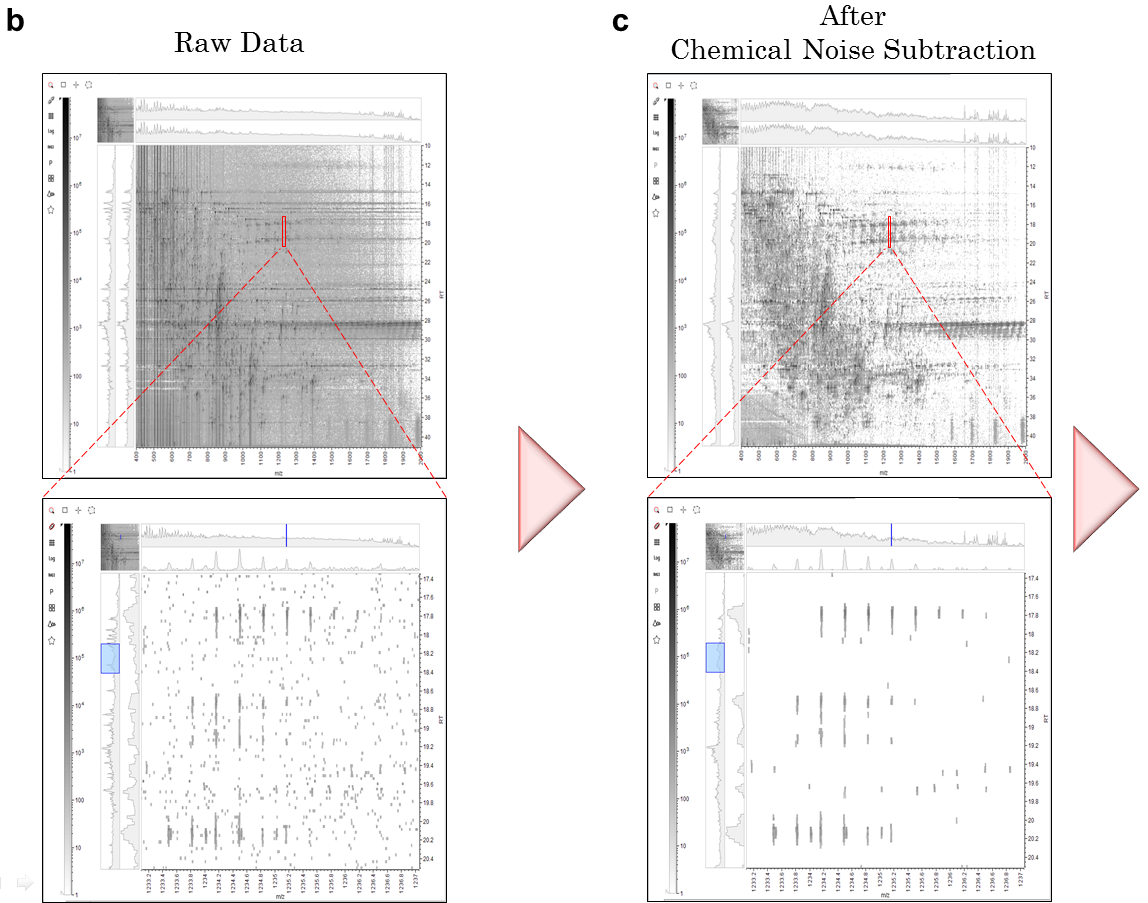


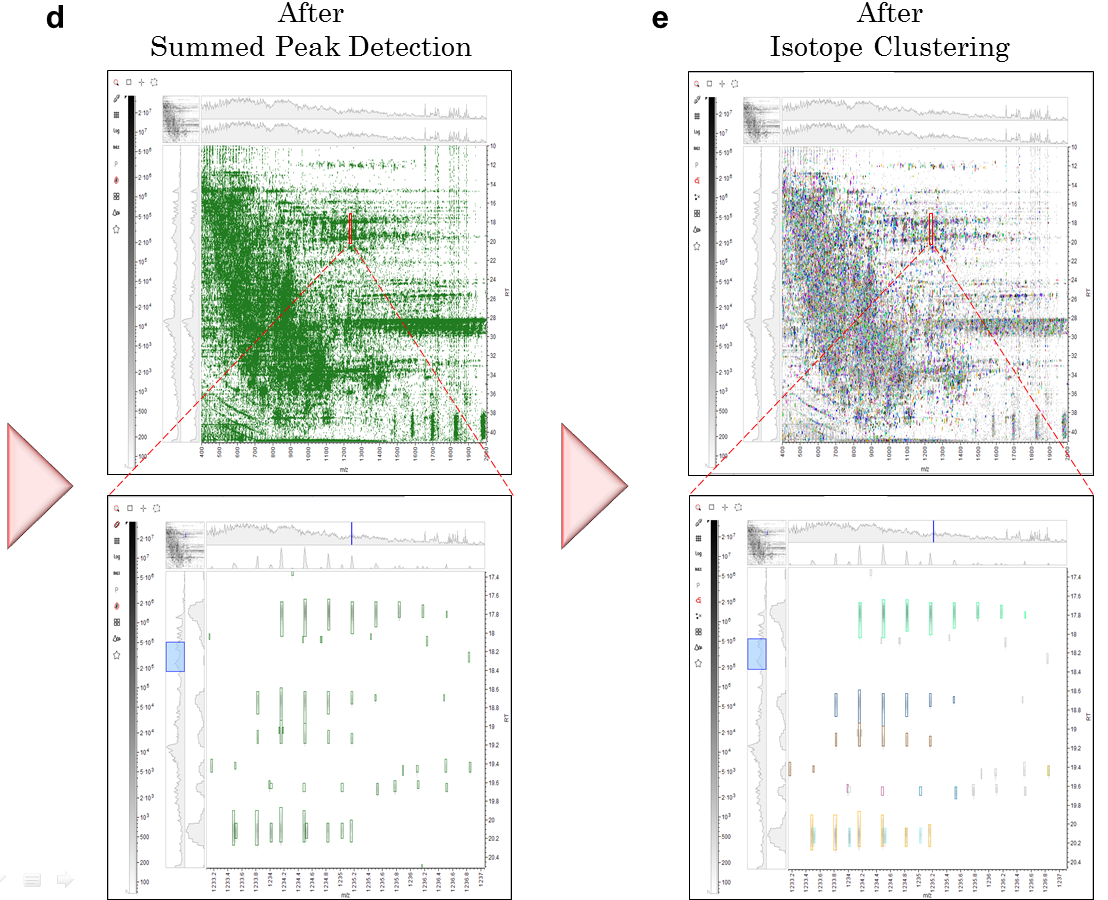


**Figure S1** | (a) Processing workflow of Expressionist RefinerMS module. Typical 2D MS chromatogram views after (b) loading .raw file, (c) chemical noise subtraction, (d) summed peak detection, and (e) isotope clustering were shown. The lower panels in (b)-(e) show magnified views of red rectangles indicated in the upper panels.

**Supplementary Figure 2**


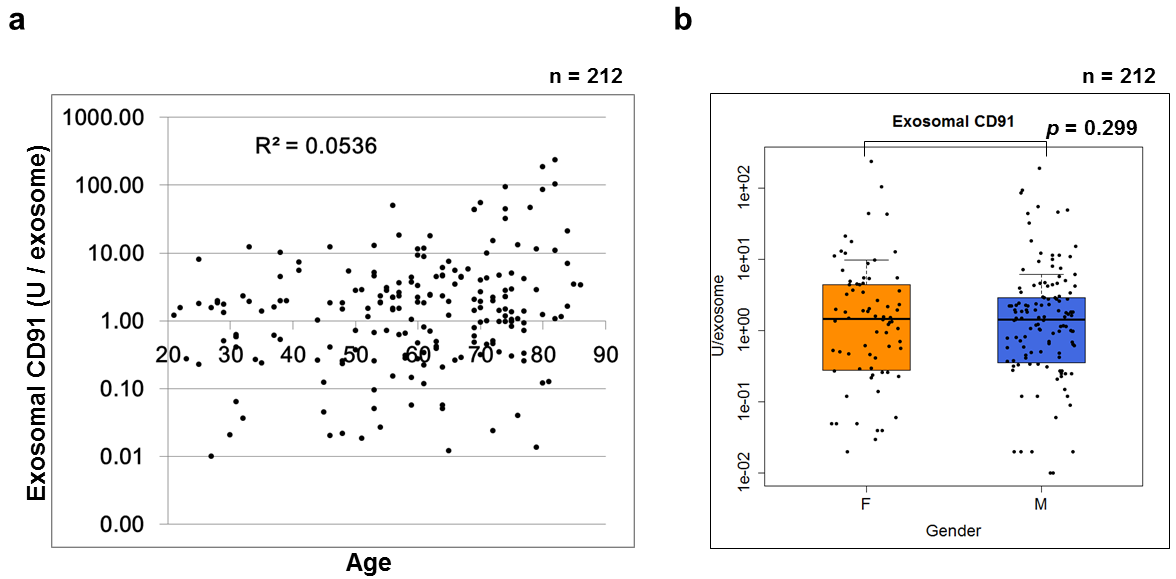


**Figure S2** | Effect of age or gender on exosomal CD91 concentrations was assessed using the validation sample set (n = 212). (a) Correlation between exosomal CD91 concentrations and age. The square of Pearson’s correlation coefficient (R^2^) is shown on the graph. (b) Gender characteristics were displayed as box plots. The *p*-value of Student’s t-test is shown. F; female, M; male.
